# Application of small molecule FPR1 antagonists in the treatment of cancers

**DOI:** 10.1038/s41598-020-74350-z

**Published:** 2020-10-14

**Authors:** Djevdet S. Ahmet, Haneen A. Basheer, Anwar Salem, Di Lu, Amin Aghamohammadi, Patrick Weyerhäuser, Andrea Bordiga, Juman Almeniawi, Sabah Rashid, Patricia A. Cooper, Steven D. Shnyder, Victoria Vinader, Kamyar Afarinkia

**Affiliations:** 1 Institute of Cancer Therapeutics, University of Bradford, Richmond Road, Bradford, BD7 1DP UK; 2 Faculty of Pharmacy, Zarqa University, PO Box 132222, Zarqa, 13132 Jordan; 3 Institut für Toxikologie, Universitätsmedizin Mainz, Gebäude 905/4. OG, Obere Zahlbacher Str. 67, 55131 Mainz, Germany; 4 Dipartimento di Scienza e Tecnologia del Farmaco, Universitá Degli Studi di Torino, Via P. Giuria 9, 10125 Torino, Italy

**Keywords:** Cancer microenvironment, Cancer prevention, Cancer therapy, Metastasis

## Abstract

The formylpeptide receptor-1 (FPR1) is a member of the chemotactic GPCR-7TM formyl peptide receptor family, whose principle function is in trafficking of various leukocytes into sites of bacterial infection and inflammation. More recently, FPR1 has been shown to be expressed in different types of cancer and in this context, plays a significant role in their expansion, resistance and recurrence. ICT12035 is a selective and potent (30 nM in calcium mobilisation assay) small molecule FPR1 antagonist. Here, we demonstrate the efficacy of ICT12035, in a number of 2D and 3D proliferation and invasion in vitro assays and an in vivo model. Our results demonstrate that targeting FPR1 by a selective small molecule antagonist, such as ICT12035, can provide a new avenue for the treatment of cancers.

## Introduction

The formylpeptide receptors, of which there are three isoforms, FPR1, FPR2 (formerly known as FPRL1 and also known as ALX) and FPR3 (formerly known as FPRL2), belong to the cell surface chemotactic GPCR-7TM family. FPRs are primarily involved in leukocyte migration to the sites of bacterial infections^1^. For example, FPR1, which is the most common of the three, is expressed on a variety of leucocytes including monocytes and macrophages^2,3^, neutrophils^2,3^, immature dendritic cells^4^, natural killer cells^5^, and T cells^6^. Leukocytes respond after their FPR1 receptors are activated by molecules which are released at the site of infection as a result of bacterial or tissue necrosis and damage^7,8^. These molecules include short chain N-formylated peptides such as N-formyl-L-methionyl-L-leucyl-L-phenylalanine (fMLF)^9,10^ and annexin A1 (ANXA1)^11,12^.

More recently, investigations have revealed that FPR1 also plays an important role in cancer. Although the expression of FPR1 in healthy, non-immune human cells is low^13–15^, it has been shown that a number of tumours express significant levels of FPR1. These include high grade glioblastoma^16–18^, neuroblastoma^19^, colon^13,20^, breast^21^, and bladder^22^ cancers. We should however note that in gastric cancer the role of FPR1 remains ambiguous. While FPR1 expression has been associated with poor prognosis in gastric cancer and correlated with tumour stage in one report^23^, another report suggested FPR1 expression suppresses gastric cancer angiogenesis and correlates with survival^24,25^. In addition, FPR1 is shown to contribute to the expansion of different tumour types. For instance, knock down of FPR1 reduces the ability of glioblastoma U87-MG tumour xenografts to grow^26^. Ye has shown that FPR1^-/-^ mice have increased survival compared with FPR^+/+^ littermates in a colorectal cancer model^20^. Finally, Vecchi and Goulart have shown that immunosuppressant cyclosporine A, which is also a weak antagonist of FPR1 (IC_50_ = 2–4 μM in calcium mobilisation assay^27^), retards tumour growth and invasiveness in a breast cancer in vivo model^21^. Whilst these studies suggest a direct role for FPR1 in tumour growth, evidence in ovarian^28^, glioma^17^ and bladder cancers^22^ as well as acute lymphoblastic leukaemia^29^, has suggested that FPR1 is also involved in resistance and recurrence.

In spite of the accumulating evidence supporting a role for FPR1 in cancer, as yet no small molecule specifically targeting FPR1 has been tested for in vivo efficacy in a tumour model. ICT12035 belongs to a class of previously published, potent and selective family of FPR1 antagonists^30^ which are now being further investigated in our lab. ICT12035 is a potent (IC_50_ = 30 nM in a calcium mobilisation assay), selective (> 100 fold versus FPR2), and non-cytotoxic (cell viability above 95 ± 5% at 100 μM) FPR1 antagonist (see Supplementary Information). Here we show that ICT12035 reduces proliferation and invasion of cancer cell lines in a number of 2D and 3D assays, and retards growth of tumours in an in vivo xenograft model.

## Results

### FPR1 is expressed in cancer cell lines, and its expression is elevated in the periphery of tumour’s hypoxic/necrotic foci

**Figure 1:**
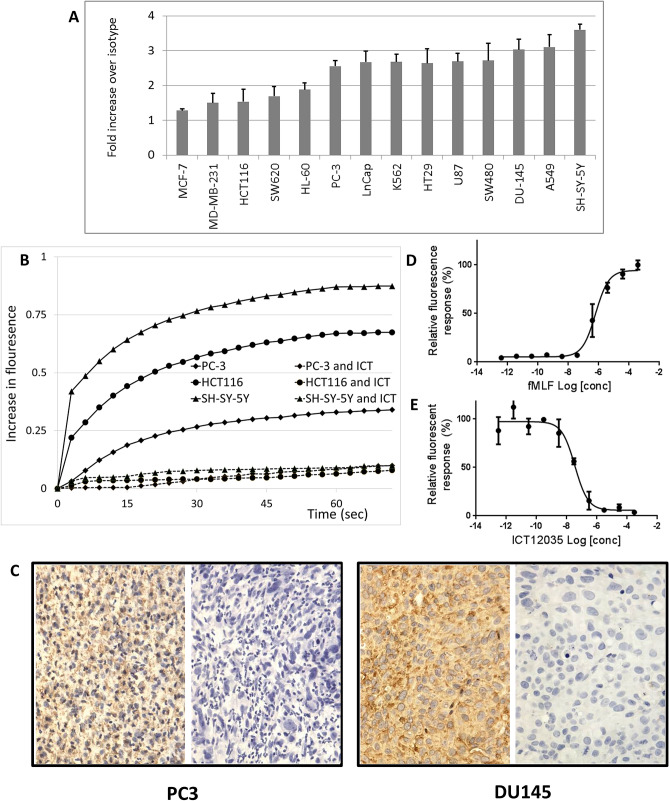
(**A**) Fold expression of FPR1 over control (isotype antibody) in different cell lines as determined by flow cytometry. (**B**) Relative fluorescence response in Ca^2+^ mobilisation (flux) assay to FPR1 agonist fMLF (100 nM) in the presence or absence of FPR1 antagonist ICT12035 (1 μM) for cell lines PC3, HCT-116 and SH-SY-5Y. (**C**) Expression of FPR1 by immunohistochemistry (IHC) in sections from PC3 and DU145 xenotransplanted tumour tissue (with and without antibody). Basophilic structure of cell was stained in blue with haematoxylin solution. (**D**) Dose response curve for U87-MG cells over concentration range of fMLF (agonist mode). (**E**) Dose response curve for U87-MG cells over concentration range of ICT12035 after treatment with 100 nM fMLF (antagonist mode).

We first determined the protein level expression of FPR1 in various cancer cell lines, using flow cytometry. Expression of FPR1 was detected in the following human cancer cell lines: U87-MG (glioblastoma), PC-3, DU-145, LnCap (prostate), SH-SY-5Y (neuroblastoma), K-562 (chronic myelogenous leukaemia), HCT-116, SW620, SW480 and HT-29 (colorectal), A549 (lung) and MDA-MB-231 (breast) (Fig. 1A). We also showed that that in addition to being present in these cell lines, the receptor is functionally active and responds to FPR1 agonist fMLF in a Ca^2+^ mobilisation assay (Fig. 1B). Furthermore, this response to fMLF is abrogated by the selective and potent FPR1 antagonist, ICT12035 (Fig. 1B, only PC-3, SH-SY-5Y and HCT-116 data are shown as examples).

Walenkamp has previously shown that expression of FPR1 in human glioblastoma cells depends on the tumour microenvironment^31^. In view of this, we also confirmed the expression of FPR1 in cancer cells grown as xenografts in mice by immunohistochemistry (IHC) (Fig. 1C, PC-3 and DU145 are shown as examples) (Fig. 1C).

We chose to study the efficacy of FPR1 antagonist ICT12035 in vitro and in vivo in the glioblastoma cell line, U87-MG. As we discussed earlier, the relevance of FPR1 as a target is shown in a number of cancers^13,16–26,28,29^. However, the expression and the relevance of FPR1 in glioblastoma is established in multiple sources^31,32^. In addition focusing on new treatment approaches to glioma is important, as it remains a particularly challenging cancer to treat. U87-MG cells do express FPR1 (Fig. 1A) and also respond to both known agonists of FPR1, fMLF (EC_50_ = 80 nM in calcium flux) (Fig. 1D) and ANXA1(2–26) (EC_50_ = 1 μM in calcium flux, in a dose dependant manner in a calcium flux assay. A dose response curve in calcium mobilisation assay (also known as calcium flux assay) showed ICT12035 antagonised the activation of FPR1 receptor by 100 nM fMLF with an IC_50_ of 30 nM (Fig. 1E).

Before using the U87-MG cell line as an in vitro model, we decided to investigate the expression of FPR1 in this cell line in 2D as well as 3D models. It is already known that 3D models such as multicellular spheroids recapitulate the conditions experienced by cancer cells within tumours’ hypoxic/necrotic regions, more closely than in 2D models, such as monolayer cells.

**Figure 2:**
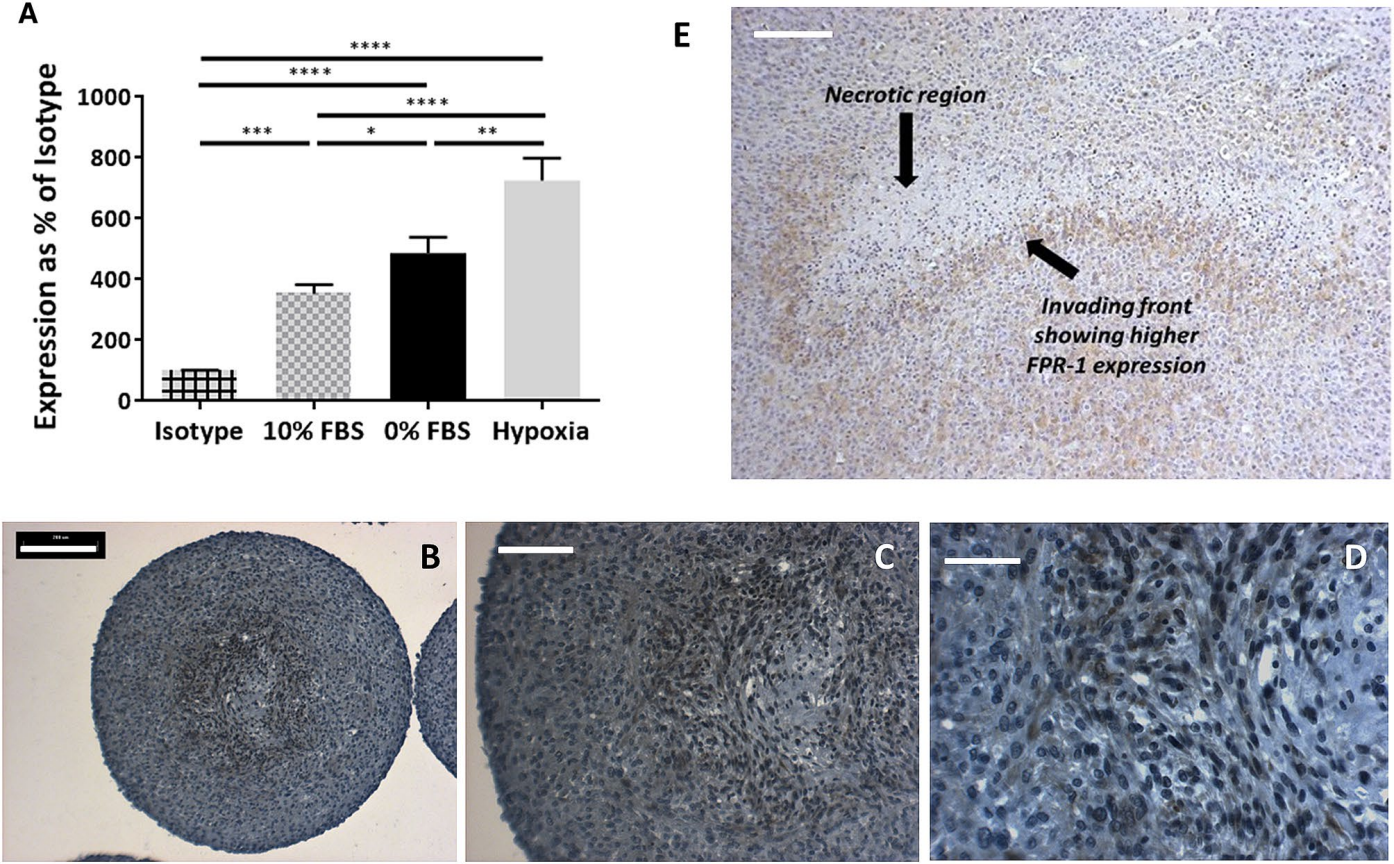
(**A**) Expression of FPR1 in U87-MG cells, cultured as monolayers, in flow cytometry over isotype control, under normal cell culture condition, serum starvation, and hypoxia (* < 0.05, ** < 0.005, *** < 0.0005, **** < 0.0001). (**B**,**D**) Expression of FPR1 by IHC in sections from a U87-MG multicellular spheroid (bar = 200 micron, 100 micron and 50 micron respectively). Basophilic structure of cell was stained in blue with haematoxylin solution. (**E**) Expression of FPR1 by IHC in sections from a U87-MG xenotransplanted tumour tissue.

Indeed, we found that the level of FPR1-expression in U87-MG cell line in 2D culture increased under hypoxic and serum deprivation stress conditions, which correlate to those experienced within the tumour’s core (Fig. 2A). Furthermore, we investigated the expression of FPR1 in sections from formalin-fixed paraffin-embedded (FFPE) spheroids of U87-MG cells. In smaller spheroids with diameter below 400 micron, the levels of expression of FPR1 remained uniform across the whole section. However, in spheroids with diameter greater than 500 micron, which did contain a necrotic core (Fig. 2B–D), we observed a higher level of FPR1 expression in the periphery of the hypoxic/necrotic core of the spheroids. To determine if the same observation applies in vivo, we examined the levels of expression of FPR1 in U87-MG cells grown as subcutaneous xenografts. Again, we observed a higher level of FPR1 expression in the periphery of the hypoxic/necrotic core of the xenoplanted tumour (Fig. 2E).

### ANXA1 is released from the hypoxic/necrotic core of 3D U87-MG multicellular spheroids

**Figure 3:**
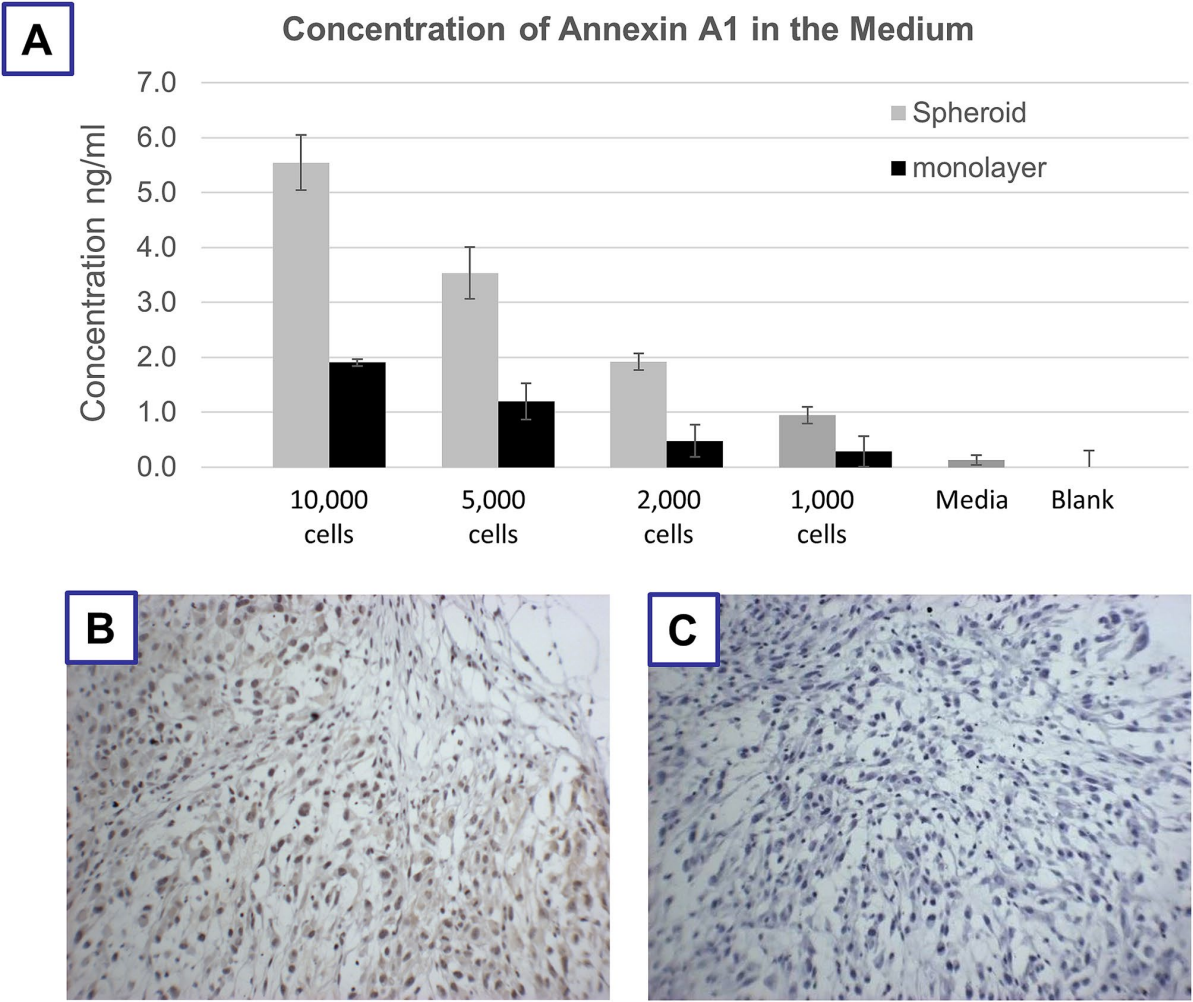
(**A**) Concentration of annexin A1 (ANXA1) released in the culture media surrounding U87-MG multicellular spheroid (grey) or over monolayer U87-MG cells (black) at different cell numbers. (**B**,**C**) Expression of ANXA1 by IHC in sections from a U87-MG xenotransplanted tumour tissue (with and without antibody). Basophilic structure of cell was stained in blue with haematoxylin solution.

It has been previously established that ANXA1 is present in the supernatant of artificially necrotised glioma cells and that ANXA1 protein within these necrotic supernatant, can stimulate tumour cell growth and migration through binding to FPR1 receptor^16,33^. In view of this and our earlier observation that FPR1 expression is elevated in the periphery of the hypoxic/necrotic core of spheroids, we wanted to determine if ANXA1 is also released from the hypoxic/necrotic core of spheroids. Using ELISA, we quantified and compared the levels of ANXA1 in the supernatant of U87-MG cells grown as 3D spheroids and as 2D monolayers (Fig. 3A). We confirmed a correlation between the quantity of excreted ANXA1 in the medium and the number of cells in both 2D (monolayer) and 3D (spheroid) systems. However, the cell culture supernatants containing spheroids contained more ANXA1 than those with the same number of corresponding cells cultured as monolayer and that difference increased for larger spheroids. The larger spheroids were subsequently isolated and shown to contain a bigger hypoxic/necrotic core. This observation strongly supports the hypothesis that ANXA1 released from the hypoxic/necrotic core diffuses out of spheroids’ core and can activate the FPR1 receptors which are in the periphery cells of the hypoxic/necrotic core. Furthermore, we confirmed the previous observation that ANXA1 is expressed in the U87-MG cells grown as subcutaneous xenografts (Fig. 3B,C).

### ICT12035 reduces fMLF-induced increases in proliferation in 2D and 3D culture

**Figure 4:**
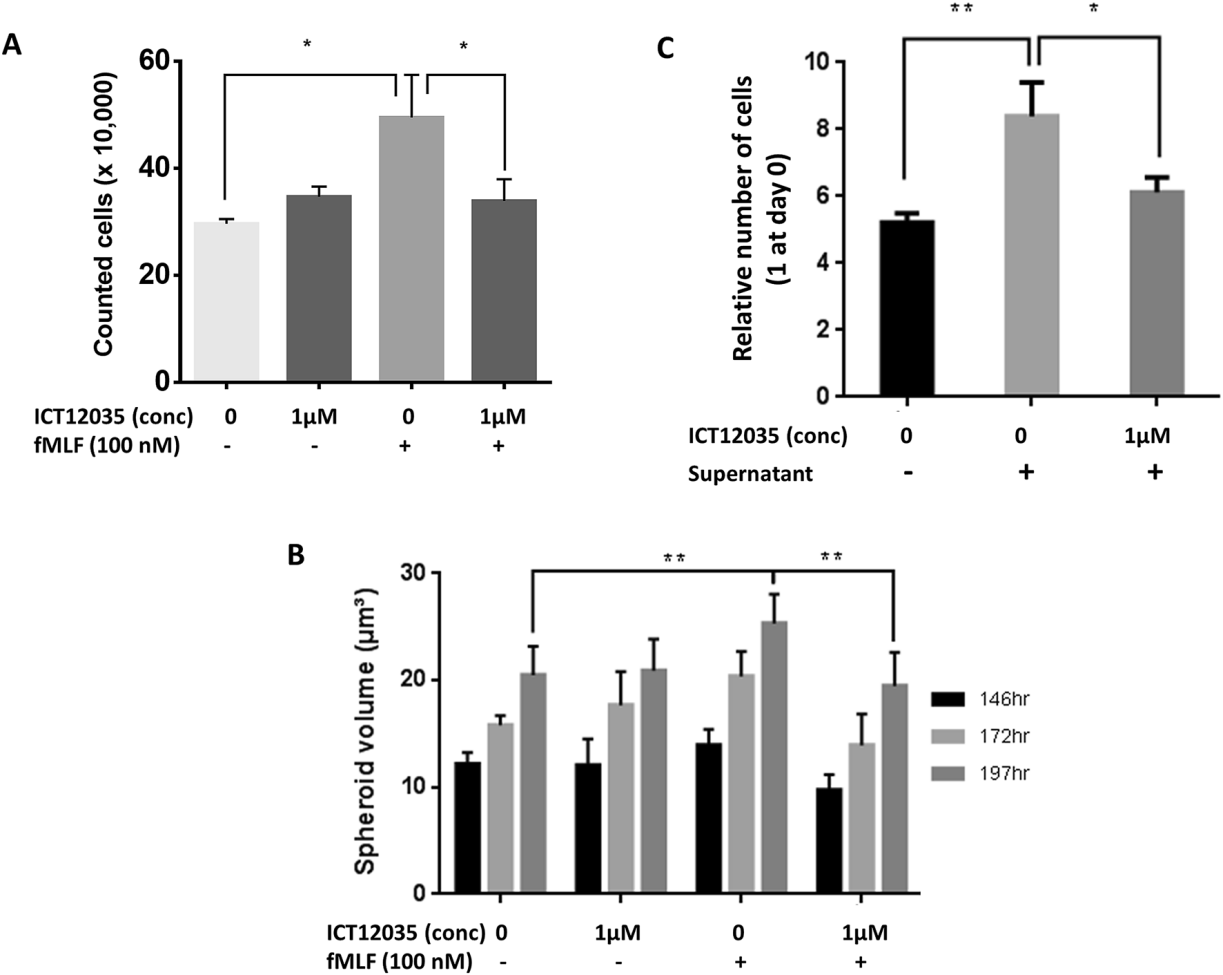
(**A**) Number of viable U87-MG cell (trypan blue exclusion) after 4 days (number of cells at t = 0 days is 10,000) (n = 8). ICT12035 is not cytotoxic and has no effect on cell viability (second coloumn). Presence of 100 nM fMLF significantly increases number of viable cells (third column) compared to control (first coloumn) (p = 0.039); however, addition of ICT12035 (1 µM) abrogates the fMLF induced increase in proliferation (p = 0.039) (* means p < 0.05) (**B**) Volume of U87-MG spheroids at 146 h, 172 h and 197 h (n = 8). ICT12035 is not cytotoxic and has no effect on spheroid growth (second coloumn). Presence of 100 nM fMLF significantly increases the spheroid growth (third column) compared to control (first coloumn) (p = 0.0031 at 197 h); however, addition of ICT12035 (1 μM) abrogates the fMLF induced increase in spheroid growth (p = 0.0014 at 197 h) (** means p < 0.005) (**C**) Relative number of U87-MG cell after 4 days (relative number of cells at t = 0 days is 1) (n = 3). Presence of supernatant significantly increases number of cells (second column) compared to control (first coloumn) (p = 0.003); however, addition of ICT12035 (1 µM) abrogates the fMLF induced increase in proliferation (p = 0.011) (* means p < 0.05; ** means p < 0.005).

We next showed that activation of the FPR1 receptor results in increased proliferation in U87-MG cells. Addition of fMLF, which is a more potent agonist of FPR1 than ANXA1, to U87-MG cells grown as monolayers increased rate of cell proliferation. However, administration of the selective FPR1 antagonist ICT12035, at the same time as fMLF, abrogated the observed increase in cell numbers (Fig. 4A).

As 2D models do not accurately recapitulate the conditions experienced by cells within tumours, we also measured growth of U87-MG multicellular spheroids as determined by increases in their volume. Pre-formed spheroids with diameters in the range of 100–120 microns (n = 8) were treated with fMLF and control, and with ICT12035 and control (Fig. 3B). The spheroids’ growth was monitored by measuring the total volume of each spheroid at 146 h, 172 h and 197 h using a Celigo Imaging Cytometer (Nexcelom)^34^. We observed a statistically significant increase in the spheroid volume at both 172 h and 197 h upon fMLF treatment compared to control. However, that increase was significantly retarded upon addition of ICT12035 (Fig. 4B).

To correlate the FPR1 mediated increase in proliferation to necrosis, we also treated U87 cells with supernatant from necrotized cells which we and others^16,33^ have shown to contain ANXA1. U87 cells were necrotized using a freeze–thaw cycle^33^. The supernatant was centrifuged to remove cell debris and was then filtered. Treatment of U87 cells with this necrotic supernatant also resulted in an upsurge in proliferation, which again could be reduced upon addition of FPR1 antagonist ICT12035 (Fig. 4C).

### ICT12035 reduces fMLF-induced increases in U87-MG cell invasion in 2D and 3D culture

**Figure 5:**
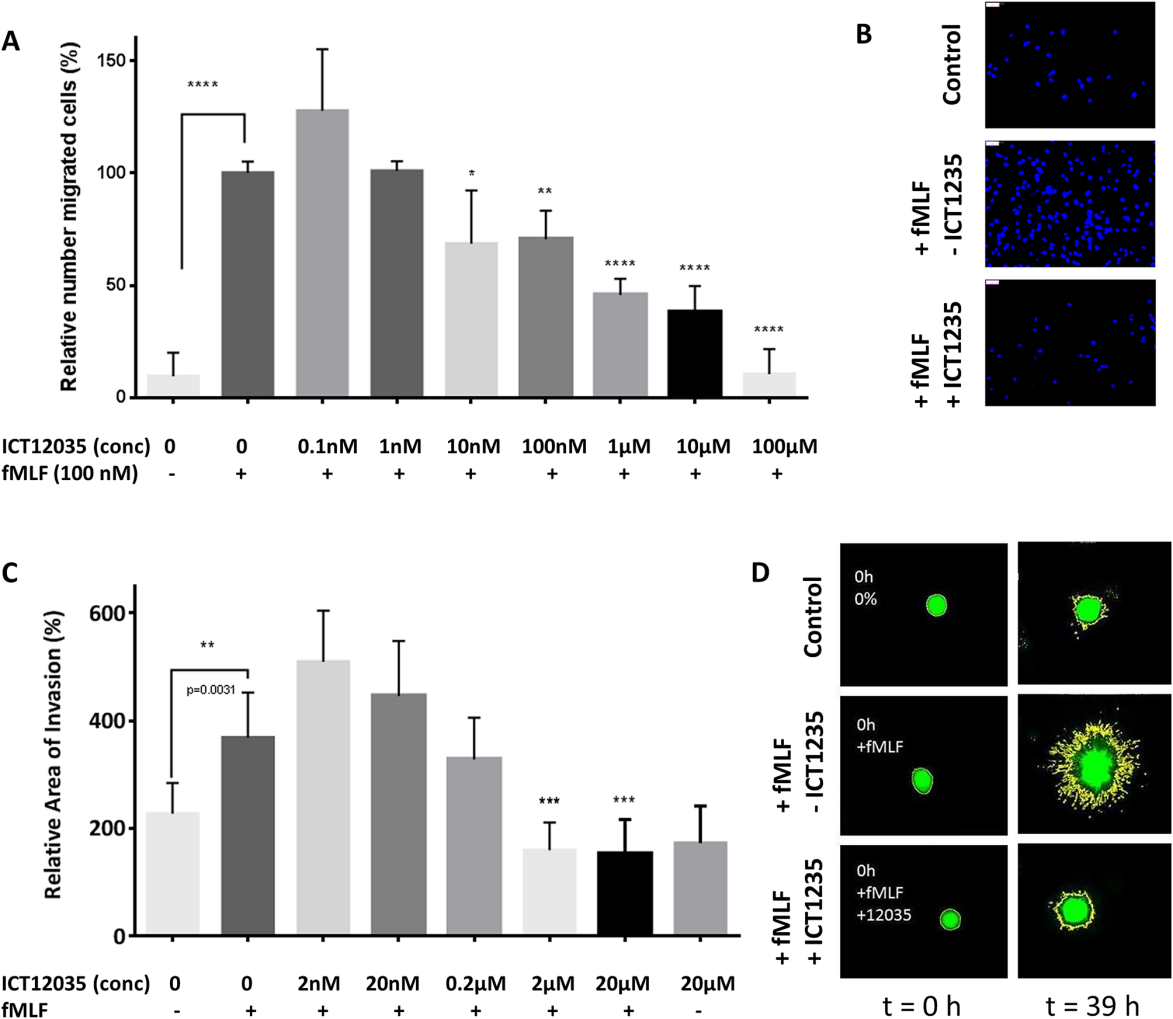
(**A**) Relative number of migrated cell in a two chamber (Boyden) assay. 100 nM fMLF significantly increases number of migrated cells (second column) compared with when no fMLF is added (p < 0.0001). This fMLF-induced increase in migrated cells is abrogated in a dose dependent manner upon addition of ICT12035 with significant reductions at 10 nM (p = 0.0398), 100 nM (p = 0.0043), 1 µM (p < 0.0001), 10 µM (p < 0.0001) and 100 µM (p < 0.0001) (* < 0.05, ** < 0.005, *** < 0.0005, **** < 0.0001). (**B**) Visual representation of differences in migration of U87-MG cells (stained with DAPI) in a Boyden assay. (**C**) Relative area of invasion for U87-MG spheroids embedded in collagen and maintained under cell culture medium. Addition of 9.14 µM fMLF to the cell culture medium significantly increases area of invasion (second column) compared with when no fMLF is added (p = 0.0031). This fMLF-induced increase in migrated cells is abrogated in a dose dependent manner upon addition of ICT12035 with significant reductions at 2 µM (p = 0.0002) and 20 µM (p = 0.0002) (* < 0.05, ** < 0.005, *** < 0.0005, **** < 0.0001). (**D**) Visual representation of differences in migration of U87-MG spheroids embedded in collagen at 0 h (left) and 39 h (right). The cells are stained with Calcein AM for ease of visualisation.

We next looked at the role of FPR1 antagonism in modulating invasiveness of U87-MG cells in 2D and 3D culture. In a Boyden assay, using inserts coated with collagen, ICT12035 retards the invasion of U87-MG cells in a dose dependant manner (Fig. 5A,B). We also investigated effectiveness of ICT12035 on invasiveness of U87-MG multicellular spheroids. The spheroids were stained with calcein for ease of detection, embedded in collagen (with and without fMLF) and analysed at 18 and 39 h by microscopy. At both time points, we observed larger cross section area for the spheroids embedded in collagen containing 100 nM fMLF. Furthermore the increase was abrogated upon addition of ICT12035 (Fig. 5C,D).

### Antagonism of FPR1 modulates growth of U87-MG xenotransplanted tumour in mice

**Figure 6:**
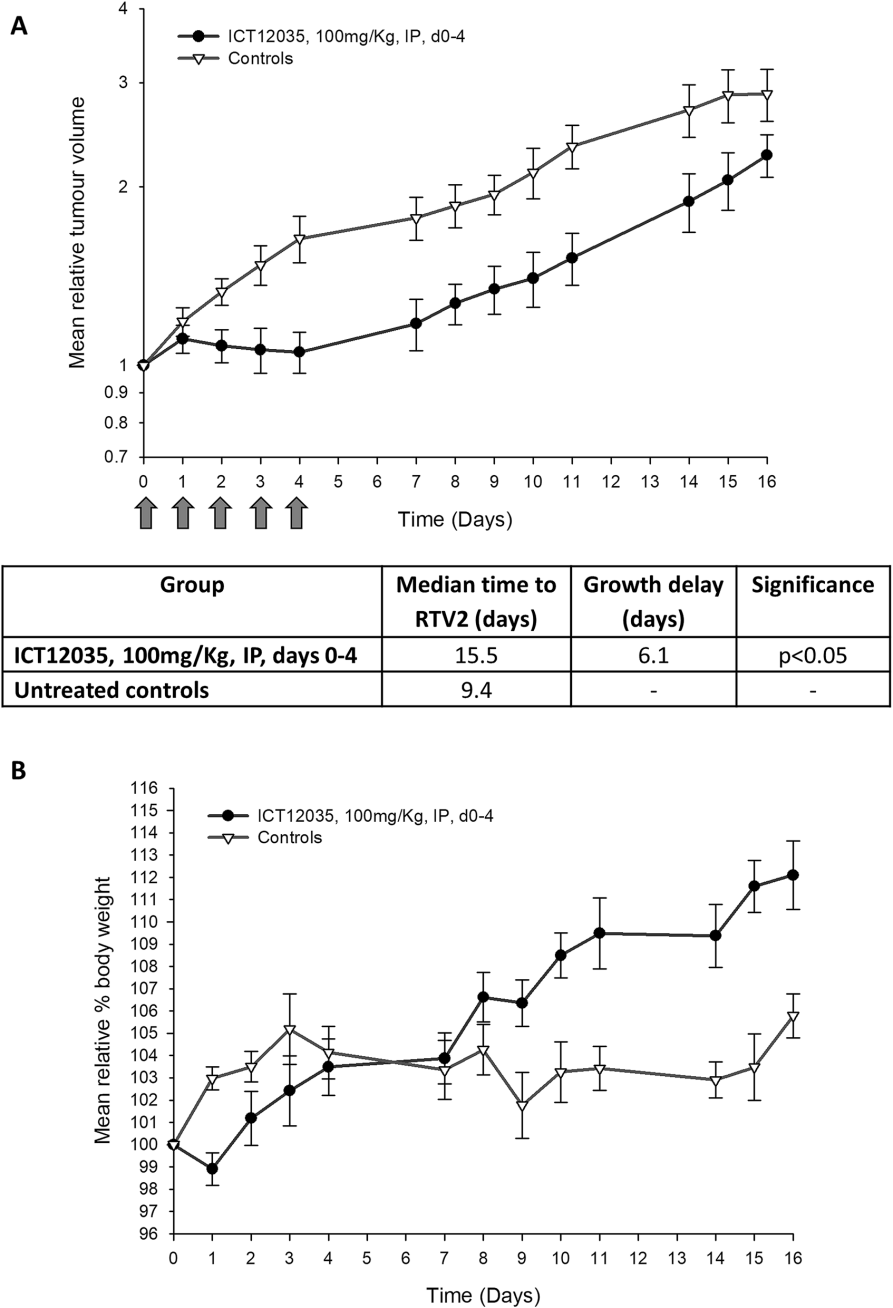
(**A**) A comparison between the growth of established xenotransplanted tumours Treatment of mice with established xenotransplanted tumours (n = 8) with five intraperitoneally administered daily doses (100 mg/Kg) of ICT12035, resulted in a 6.1 days (p < 0.05) delay in tumour growth compared with control; (**B**) Administration of ICT12035 had no adverse effect on mice and resulted in no weight loss over the experiment period.

Finally, we used a subcutaneous U87-MG xenograft model to assess in vivo efficacy of ICT12035. Prior to the efficacy experiments, non-tumour bearing mice (n = 4) were treated with escalating doses of ICT12035 and we observed no toxicity upto a maximum dose of 100 mg/Kg. Therefore, the drug was administered at 100 mg/Kg dose for five consecutive days to tumour bearing mice and the tumour growth was monitored compared to a control group (n = 8 for both). No signs of toxicity were evident, and encouragingly, we observed a six day delay (p < 0.05) in further growth of the tumours (Fig. 6).

## Materials and methods

### Cells and reagents

All cell lines were obtained from either the European Collection of Cell Cultures (ECACC) or American Type Cell Culture (ATCC) and maintained in recommended media supplemented with 10% (v/v) foetal calf serum, 1 mM sodium pyruvate and 2 mM L-glutamine; at 37 °C in an atmosphere of 5% CO_2_. All cell lines are authenticated in the past three years. ICT12035 was prepared in the laboratories of the Institute of Cancer Therapeutics, University of Bradford.

### Flow cytometry

Two Eppendorfs containing 30–50 × 10^4^ cells each were treated with 20 μL of anti-hFPR1 (PE-conjugated, R&D system, FAB3744P) and mouse IgG_2A_ isotype control (PE-conjugated, R&D system, IC003G). Both tubes were incubated in the dark at 0 °C for 45 min. After washing with 1% BSA in PBS, the cells were analysed using a BD Accuri C6 plus flow cytometer.

### Formation of spheroids

U87-MG cells (1 × 10^6^ cells/ml) were seeded into a spinner flask (F7690, Techne, Bibby Scientific Limited, Staffordshire, UK) containing 150 mL of RPMI-1640 containing 10% FCS (v/v), and placed on a magnetic stirrer plate (MCS-104S, Techne, Bibby Scientific Limited, Staffordshire, UK). The mixture was stirred at a rate of 55 rpm for 5 days.

### Immunohistochemistry

The 5 micron thick sections from FFPP blocks of xenoplanted tumour tissue or spheroids were incubated with the rabbit polyclonal FPR1 primary antibody (Abcam, ab113531).

### Annexin A1 quantification using ELISA

This was carried out using a sandwich enzyme-linked immunosorbent assay (ELISA) kit for human Annexin A1 purchased from Abcam (ab222868).

### Preparation of necrotic supernatant

A suspension of about 55 × 10^4^ cells/mL of U87-MG cell in PBS was subjected to 7 cycles of rapid freezing at − 80 °C and thawing at room temperature. The mixture was centrifuged and the supernatant was filtered to remove any debris.

### Cell viability assay using MTT

Into each well of the 96 well plates, 180 µL of U87-MG cell suspension (5 × 10^4^ cells/mL) were seeded. 20 µL of either PBS (as control) or ICT12035 solution in increasing concentrations were also added. Readings for each concentration were carried out in quadruplicate. Plates were incubated at 37 °C, 5% CO_2_, for 96 h. Then, 20 µL of a 5 g/L solution of 3-(4,5-dimethyl-2-thiazolyl)-2,5-diphenyl-2H-tetrazolium bromide (MTT) (Alfa Aesar, L1193) in water was added. The plate was incubated at 37 °C, 5% CO_2_ for 4 h after which, the solution was carefully removed and DMSO (150 µL) was added to dissolve the formazan crystals. The absorbance of each well was measured at 540 nm using a Multiscan Ex 96 well microplate reader (Thermo Electron Corporation, United Kingdom). The experiment was repeated in triplicate (n = 3).

### Cell viability assay using cell counting

Into four wells of a 6-well plate, 1980 µL of U87-MG cell suspension (5 × 10^4^ cells/mL) were added. To one well was added 20 µL of PBS (control plate); to the second well was added 20 µL of fMLF in PBS to achieve an in well concentration of 100 nM; to the third well was added 20 µL of ICT12035 in PBS to achieve an in well concentration of 1 µM; and to the fourth well was added 10 µL of each ICT12035 and fMLF in PBS to achieve an in well concentration of 1 µM and 100 nM respectively. After 5 days, each well was treated with trypsin/EDTA solution until cells were fully detached, and viable cell numbers were counted (trypan blue exclusion). The experiment was repeated in triplicate (n = 3).

### Spheroid growth assay

Spheroids were generated by adding 200 μL of a 2500 cell/mL U87-MG cell suspension into each well of an ultralow adherence 96 microtiter plate (ULA-96U from Nexcelom). Spheroids were formed after 48 h. The spheroids were treated once every 24 h with either PBS (as control), 100 nM fMLF, 1 μM ICT12035 (in well concentration) or both fMLF and ICT12035. The increase in the volume of spheroids were monitored for 9 days using a Celigo Imaging Cytometer using instruments software^34^.

### Calcium mobilisation assay

This was carried out by an adaptation of the recommended protocol^35^. 10 × 10^4^ U87-MG cells were seeded into each well of a Corning tissue culture treated 96-well black polystyrene assay plate (VWR, 734–1609). After 24 h, the growth medium was removed and replaced with 100 µL of the dye loading solution (Molecular Probes Fluo-4 NW (no wash), Invitrogen F36206). The plates were incubated at 37 °C for 30 min. 20 μL of a given concentration of the antagonist in assay buffer, or plain assay buffer as control was added to each well and the plate was incubated at 37 °C for another 30 min. The plate was transferred into a Fluoroskan Ascent FL instrument (ThermoScientific) and the fluorescence in response to the addition of 20 μL of 100 nM fMLF (in well concentration) was measured at 37 °C (Ex 485 nm, Em 538 nm). IC_50_ is calculated as the concentration of the antagonist required to half the maximal response to fMLF. The experiment was repeated in triplicate (n = 3).

### Chemotaxis assay

Was performed in 24-well chemotaxis chamber inserts (Corning, Product code: 3415). The filters were coated with 50 μg/mL collagen type I solution from rat tail (Sigma, C3867). To the lower chamber was added 600 μL of RPMI-1640 (no serum) containing either no agonist (control) or fMLF (1.62 µM). To the upper compartment were added 150 μL of U87-MG cells (6.7 × 10^5^ cells/mL) containing the appropriate concentration of ICT12035. After 16 h, the medium was removed and replaced with ethanol to fix the cells. Using a cotton bud, the non-migrated cells in the top compartment were gently removed from the membrane. The membrane was allowed to air dry and then was removed using a scalpel. The membrane was mounted onto a slide using mounting medium containing DAPI (Vector Labs, VECTASHIELD hard set mounting medium with DAPI). The mounted slides were viewed under a fluorescent microscope with a LeikaDM2000 camera attached. Six images from varying fields at a 20 × magnification were taken. The images were analyzed using ImageJ software ^36^. The result was given as the percentage average area of the image taken up by the DAPI stained (blue) nuclei. The experiment was repeated in triplicate (n = 3).

### Spheroid invasion assay

1 µL of Calcein AM (4 mM in DMSO) was added to U87-MG cell suspended in 10 mL RPMI-1640 containing 10% v/v foetal calf serum. After 30 min incubation at 37 °C, the cell suspension was centrifuged, and the supernatant discarded. The cells were resuspended in culture medium containing methyl cellulose (v/v 20%) to a concentration of 8 × 10^4^ cells/mL. The lid of a 60 mm tissue culture dish was inverted, and 5 mL of PBS added to the bottom of the dish. A multi-channelled pipette was used to deposit 25 µL drops (2000 cells) to the base of the lid. The lid was then inverted and placed onto the PBS-filled bottom chamber. The dish was incubated at 5% CO_2_ and 37 °C for 24 h or until spheroids had formed. Ice cold aqueous sodium hydroxide (1 M, 108.33 µL) was added to an ice cold mixture of 5 × PBS (2 mL), distilled water (3.56 mL) and collagen I (from rat tail, Corning, 354236) (4.33 mL). This solution was equally divided between different Eppendorfs, and either PBS (control), fMLF to a final concentration of 9.14 μM, ICT12035 to a final concentration of 20 μM or fMLF (9.14 μM) and various concentrations of ICT12035 were added. The wells of a 96-well glass-bottomed black plate (Thermofisher, 160376) were covered with 40 µL of each of the three collagen mixtures which after warming to room temperature formed a gel. Single spheroids added onto each well containing a layer of collagen gel. Another 40 µL Collagen mix was gently added over the top of each spheroid to ensure the spheroid was encased in collagen gel. On top of the final layer of collagen gel was added serum free RPMI-1640 (50 µL) to prevent the spheroids/gel from drying out. Images were taken at 0 h and 39 h using a LumaScope 500 and the green fluorescence filter to detect the preloaded Calcien AM within the cells. The images were analysed using ImageJ software^36^.

### In vivo efficacy of ICT12035

All procedures were carried out under Project Licence PPL 40/3670 issued by the UK Home Office according to government legislation, following approval of the work by the local Animal Welfare Ethics Review Board at the University of Bradford, and in accordance with the UK National Cancer Research Institute Guidelines for the Welfare of Animals^37^. Balb/c immunodeficient nude mice (Envigo, Loughborough, UK), between the ages of 6 and 8 weeks, were used. Under brief general inhalation anaesthesia 2 to 3 mm^3^ fragments of U87-MG human tumor xenografts were implanted subcutaneously in the abdominal flank of the mice. Once tumour volumes reached approximately 32 mm^3^ (as measured by callipers), this was designated as Day 0, and mice were randomised into two groups (n = 8). One group received ICT-12035 administered intraperitoneally at 100 mg/kg dose on days 0, 1, 2, 3 and 4. For comparison, the control group was left untreated. Tumour volume using callipers and animal body weight were recorded through the experiment and normalised to the respective volume on the initial day of treatment (day 0). Mann–Whitney U tests were conducted to determine the statistical significance of any differences in growth rate (based on tumour volume doubling time) between control and treated groups.

## Discussion

Dysregulated and improper vascularisation during tumour growth reduces access to oxygen and nutrients in regions within the tumour resulting in the formation of hypoxic/necrotic foci within tumours. This tumour necrosis plays a multifaceted and complex role in many tumours^38–40^. In particular, necrotised cells release many of their cytoplasmic molecules, which includes annexin A1 (ANXA1) protein, into the extracellular milieu^30,41–44^. The release of these proinflammatory molecules recruits leukocytes to the tumour microenvironment. Interestingly, the inflammatory response within tumours appears to both help and hinder tumour expansion^45^.

In addition, ANXA1 appears to have a distinct tumour promoting effect by binding and activating FPR1, the expression of which is shown to be elevated in many tumours^13,16–22^. The activation of FPR1 by ANXA1 promotes angiogenesis, proliferation, and invasion in a number of cancers^13,46^. It follows that inhibiting the activation of FPR1 by ANXA1, may have a potential role as a therapy against a number of cancers. However, to date no small molecule FPR1 antagonist has ever been investigated as a potential cancer therapy. ICT12035 is a potent and selective small molecule FPR1 antagonist^30^. We set out to provide the proof of the principle that a small molecule FPR1 antagonist, such as ICT12035, can have a potential as a therapy for cancers such as glioma.

A substantial body of work has already shown that the expression of FPR1 is elevated in glioma, and that the expression correlates with the stage of tumours^16^. More interestingly, Walenkamp has shown that the expression is enhanced under conditions within the tumour microenvironment^32^. Our observation (Fig. 1) that FPR1 expression is particularly elevated in the periphery of the necrotic/hypoxic core, both in a 3D spheroid model and in xenografted tissue, strengthens the correlation of FPR1 axis to tumour necrosis. In tissue, the so called pseudopalisades are known to be rich in HIF-1α, carbonic anhydrase IX and MMPs and may be considered an invading front of aggressive glioma tumours^47^. Although we have not yet investigated the drivers for the elevation of FPR1 expression in the periphery of the tumour core, we can speculate that may be partly due to hypoxia. Hypoxia is already known to elevate expression of another chemotactic receptor, CXCR4, in glioma^48^. Furthermore, we and others^49^ have shown that release of ANXA1 also correlates to hypoxia/necrosis which in turn may enhance FPR1 expression through an autocrine loop^21^.

In assessment of the efficacy of ICT12035, we opted for the use 3D in vitro models such as spheroids. 3D in vitro models are generally more advantageous and relevant than 2D models, and provide more reliable data^50^. Indeed, ICT12035 retards both proliferation and invasion of U87 spheroids as well as monolayers (Figs. 3 and 4). Furthermore, to show the relevance of necrosis to the FPR1-mediated expansion of gliomas, we used ICT12035 to retard increases in both proliferation and invasion of U87 spheroids as well as monolayers by necrotic U87 supernatants.

Finally, treatment of mice bearing xenotransplanted tumours with ICT12035 over 5 days, completely arrested growth of the tumours during the treatment period, but as expected, had no toxic side-effect on the mice.

Our further preliminary investigations, which we will report in due course, reveal similar effects of FPR1 activation in other cancer types. This clearly demonstrates the usefulness of FPR1 antagonists, particularly in treatment aggressive tumours. Furthermore, it is known that necrosis results from a number of treatment modalities in cancers and is hypothesised to contribute to poor clinical outcomes of current therapies^39,51,52^. This raises an interesting hypothesis that combining current therapies with antagonism of FPR1, at least in parts, may contribute to making them more efficacious. Therefore, further investigation of ICT12035, or other FPR1 antagonists, can open opportunities for more efficient treatment for cancers, including glioma, in due course.

## Supplementary information

Supplementary information.

## Data Availability

The data generated during and/or analysed during the current study are available from the corresponding author on reasonable request.
