# Supplementary material for: Application of small molecule FPR1 antagonists in the treatment of cancers
Source: Sci Rep. 2020 Oct 14;10:17249. doi: 10.1038/s41598-020-74350-z (PMC7560711; doi:10.1038/s41598-020-74350-z)
Supplement: Supplementary file 1 — Supplementary information. [file 41598_2020_74350_MOESM1_ESM.pdf]

Supplementary Information

Application of Small Molecule FPR1 Antagonists in the Treatment of Cancers: A Proof of Concept Study

Djevdet S. Ahmet, Haneen A. Basheer, Anwar Salem, Di Lu, Amin Aghamohammadi, Patrick Weyerhäuser, Andrea Bordiga, Juman Almeniawi, Sabah Rashid, Patricia A. Cooper, Steven D. Shnyder, Victoria Vinader, and Kamyar Afarinkia*
Correspondence to: k.afarinkia@bradford.ac.uk

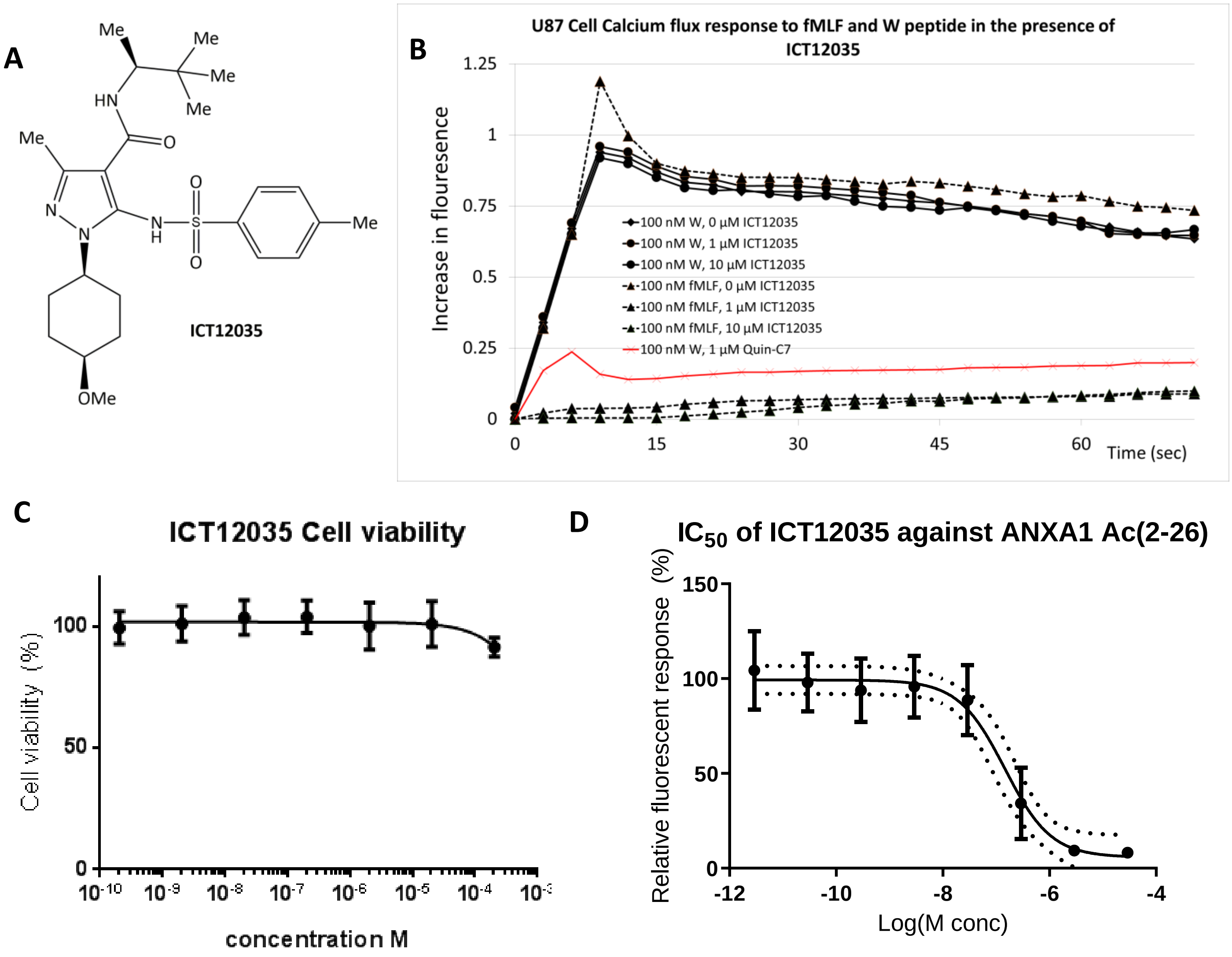

Figure S1: (**A**) Chemical structure of ICT12035 (see ref 32). (**B**) Relative fluorescence response in Ca^{2+} mobilisation (flux) assay to FPR1 agonist WKYMVm (100 nM) in the presence or absence of FPR1 antagonist ICT12035 (0, 1 and 10 μM) in U87-MG cell line. The red line represents relative fluorescence response in the presence or absence of FPR2 antagonist Quin-C7[ref] (1 μM). Average of three experiments. (**C**) U87-MG Cell viability after treatment with range of concentrations of ICT12035 shows the compound is not cytotoxic at concentrations used for in vitro experiments. Average of three experiments. (**D**) Dose response curve for U87-MG cells over concentration range of ICT12035 after treatment with 100 nM ANXA1 Ac(2-26) (antagonist mode). Average of three experiments. Dotted lines show 95% confidence limits.

[ref] = Zhou C, Zhang S, Nanamori M, Zhang Y, Liu, Q, Li N, Sun M, Tian J, Ye PP, Cheng N, Ye RD, Wang MW "Pharmacological characterization of a novel nonpeptide antagonist for formyl peptide receptor-like 1" *Mol Pharmacol.* **2007**, 72(4), 976-983. A correction appeared in *Mol Pharmacol.* **2007**, 72(5), 1391.
